# CEL-Seq2: sensitive highly-multiplexed single-cell RNA-Seq

**DOI:** 10.1186/s13059-016-0938-8

**Published:** 2016-04-28

**Authors:** Tamar Hashimshony, Naftalie Senderovich, Gal Avital, Agnes Klochendler, Yaron de Leeuw, Leon Anavy, Dave Gennert, Shuqiang Li, Kenneth J. Livak, Orit Rozenblatt-Rosen, Yuval Dor, Aviv Regev, Itai Yanai

**Affiliations:** Department of Biology, Technion – Israel Institute of Technology, Haifa, Israel; Department of Developmental Biology and Cancer Research, The Institute for Medical Research Israel-Canada, The Hebrew University-Hadassah Medical School, Jerusalem, Israel; Klarman Cell Observatory, Broad Institute of Harvard and MIT, Cambridge, MA 02142 USA; Department of Biology, MIT, Cambridge, MA 02139 USA; Howard Hughes Medical Institute, Department of Biology, Massachusetts Institute of Technology, Cambridge, MA 02140 USA; Fluidigm Corporation, 7000 Shoreline Court, Suite 100, South San Francisco, CA 94080 USA

## Abstract

Single-cell transcriptomics requires a method that is sensitive, accurate, and reproducible. Here, we present CEL-Seq2, a modified version of our CEL-Seq method, with threefold higher sensitivity, lower costs, and less hands-on time. We implemented CEL-Seq2 on Fluidigm’s C1 system, providing its first single-cell, on-chip barcoding method, and we detected gene expression changes accompanying the progression through the cell cycle in mouse fibroblast cells. We also compare with Smart-Seq to demonstrate CEL-Seq2’s increased sensitivity relative to other available methods. Collectively, the improvements make CEL-Seq2 uniquely suited to single-cell RNA-Seq analysis in terms of economics, resolution, and ease of use.

**Electronic supplementary material:**

The online version of this article (doi:10.1186/s13059-016-0938-8) contains supplementary material, which is available to authorized users.

## Background

Single-cell transcriptomics is a transformative method with tremendous potential to illuminate the complexities of gene regulation. Single-cell RNA-Seq was first introduced by Tang et al. [1], using a polyT primer with an anchor sequence to select for the cell’s mRNA. After polyadenylation of the resulting cDNA, a second polyT primer with a different anchor is used to obtain double stranded DNA, which is then PCR-amplified. Each sample is individually converted to a library for sequencing. The STRT method introduced early barcoding at the reverse transcription stage [2], thereby enabling highly-multiplexed analyses, and adapted a template switching mechanism based on the ability of the reverse transcriptase to tag the end of the cDNA [3], eliminating the need for the polyadenylation reaction. Smart-Seq [4, 5] used the same template switching mechanism as STRT, but without the early barcoding. Each sample is processed individually, and the reaction was optimized for full transcript sequencing.

The CEL-Seq [6] method is the first method to use in vitro transcription (IVT) for the amplification, thereby eliminating the requirement for a template-switch step which is thought to reduce efficiency. We use early barcoding, enabling highly-multiplexed analysis, and 3′ end tagging enabling accurate estimation of expression levels without having to account for gene length and with fewer sequencing reads required. Here we introduce CEL-Seq2, which has been optimized for higher sensitivity, less hands-on time, and lower price. We show that CEL-Seq2 works well on different platforms, and compare it to previously published methods.

## Results and discussion

### CEL-Seq2 is optimized for higher sensitivity

**Fig. 1:**
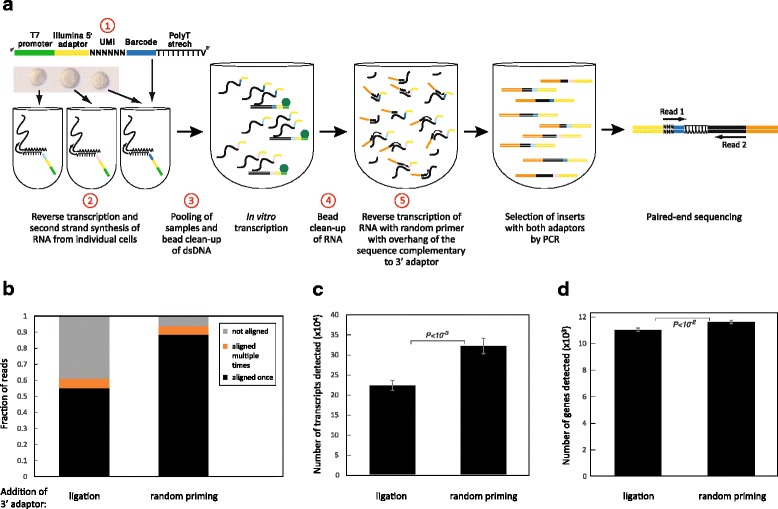
Changes introduced to the protocol. **a** An outline of the CEL-Seq2 method is shown with the steps modified from the original CEL-Seq indicated in *red*. **b** Distribution of the reads in two libraries prepared with or without ligation from the same amplified RNA of ten replicates of 100 pg clean RNA. **c**, **d** Number of transcripts (**c**) and genes (**d**) detected. Error bars indicate standard error

Recent adaptations of CEL-Seq [7, 8] integrated unique molecular identifiers [9, 10] (UMI) into the CEL-Seq primer, enabling each reverse-transcribed mRNA to be counted precisely once. Estimating CEL-Seq’s sensitivity as the fraction of ERCC spike-ins [11] transcripts detected using such UMIs, we and others [7] computed CEL-Seq’s efficiency at ~6 %. This may be an underestimate, however, because comparison with smFISH indicates threefold higher sensitivity [7]. Seeking to improve CEL-Seq’s efficiency, we introduced several changes, summarized in Fig. 1a.

First, we sought to increase the efficiency of the reverse transcription (RT) reaction by shortening the CEL-Seq primer from 92 to 82 nucleotides, despite the addition of six UMI nucleotides. This was done by reducing the length of the barcode from eight to six nucleotides, as well as shortening the T7 promoter and the Illumina 5’ adaptor. Use of the shortened primers indeed improved the sensitivity to 10.6 %, detecting more transcripts (Additional file 1: Figure S1). In this analysis on 100 pg of RNA, the number of detected genes also increased, though not significantly (Additional file 1: Figure S1a), which likely reflects that most of the additionally identified transcripts are of genes already detected using the longer primer.

We next optimized the conversion of RNA to dsDNA by testing alternative commercially available reverse transcriptases for cDNA synthesis and polymerases for second-strand synthesis. We found that SuperScript II for the RT step (Additional file 1: Figure S1d) provided a major improvement. For second strand synthesis, the differences were less pronounced, but the polymerase and other components from the SuperScript II Double-Stranded cDNA Synthesis Kit were better than its competitors (Additional file 1: Figure S1e). We also modified our method of dsDNA and aRNA clean-up from column to beads, which provided a threefold gain in yield (Additional file 1: Figure S1d). While CEL-Seq was originally implemented using the Ambion MessageAmp II aRNA Amplification Kit, these changes led to a kit-free (and therefor cheaper) process because the reagents of the SuperScript II Double-Stranded cDNA Synthesis Kit may be purchased separately.

### Ligation-free library preparation improves read mapping

In the original CEL-Seq protocol, the aRNA is converted to a library compatible with Illumina sequencing by ligating the second adaptor [6]. Following conversion to cDNA with RT, a few PCR cycles completed the attachment of Illumina adaptors. The ligation step is not efficient, however, and introduces primer dimers that interfere with sequencing. CEL-Seq2 remedies this by inserting the Illumina adaptor directly at the RT step as a 5′-tail attached to a random hexamer (Fig. 1a, change 5), thus eliminating the ligation step. We prepared a library introducing the Illumina adaptor by ligation or using the random hexamer (no ligation) from the same amplified RNA. Sequencing of the “no ligation” libraries yields 93.8 % mapping of the reads with barcodes, an improvement from 60.9 % in the ligated library (Fig. 1b). This modification also led to the identification of more genes and transcripts (Fig. 1c, d), suggesting that the removal of the ligation step significantly increased the sensitivity. To control for sequencing depth, we sub-sampled 300,000 reads from each sample, and obtained similar results (Additional file 2: Figure S2a, b). In addition, this modification reduces the hands-on time and cost of library preparation and it alleviates the need for Illumina’s TruSeq Small-RNA kit.

### CEL-Seq2 is compatible with different platforms

Our implementation of CEL-Seq for individual tubes is easily scaled-up to plates and could be performed with robotic liquid handlers. To further improve the efficiency, we sought to implement CEL-Seq2 on the Fluidigm C1, a nanoliter automatic microfluidic instrument. After capture of individual cells, the C1 loads the individually barcoded CEL-Seq2 primers from the outlet wells, lyses the cells chemically (rather than our traditional lysis by freezing), and performs an RT reaction followed by second-strand synthesis. In place of a cDNA clean-up, the second strand enzymes are heat inactivated. IVT then occurs for each sample individually. The amplified RNA is harvested from the C1 chip and pooled to a single sample from which a library is prepared.

**Fig. 2:**
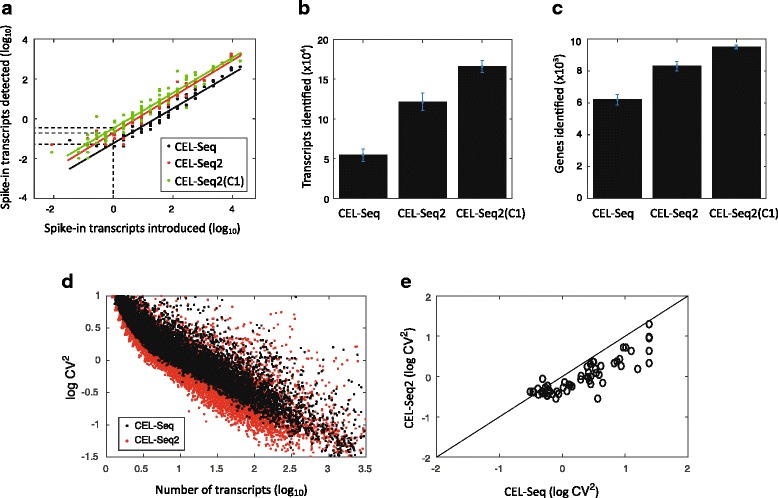
Performance of CEL-Seq2 compared to the original CEL-Seq. In total, 24, 20, and 72 single fibroblasts were analyzed by CEL-Seq, CEL-Seq2, and CEL-Seq2(C1), respectively. **a** For each of the 92 ERCC spike-ins the average observed and expected molecules across the examined cells is plotted. The lines were fitted using a linear model and the efficiency was computed as the y-intercept. **b** Comparing the mean number of transcripts identified per cell between the methods. **c** Comparing the mean number of genes identified per cell between the methods. **d** Coefficient of variation for the original CEL-Seq and CEL-Seq2. Each dot indicates a gene’s squared coefficient of variation. **e** A comparison between the squared coefficients of variation for the detected ERCC spike-ins. Error bars indicate standard error

We performed CEL-Seq2 both manually and using the C1 on mouse fibroblast cells carrying a CyclinB1-GFP fusion reporter [12] and compared this to data obtained using the original protocol (with the single modification that the primers contained UMIs). We processed 24 cells using the original CEL-Seq protocol and 20 cells using CEL-Seq2, as well as cells loaded on the C1 (we had 72 single cells captured, Additional file 3: Table S1). Spike-ins were added to each cell allowing us to compare the efficiencies of transcript detection by fitting a linear relationship on the log-log plot (Fig. 2a). The intersect with the y-axis provides a measure of the efficiency and brings us to 19.7 %, relative to 5.8 % for CEL-Seq. On the C1 an even higher efficiency is obtained of 22 %. These efficiencies are based on the spike-ins and are probably an under-estimation of the true efficiency.

The increased sensitivity of CEL-Seq2 is observed both in terms of the increased detection of transcripts and genes. CEL-Seq2 identifies twice as many transcripts per cell compared to the original protocol (Fig. 2b) and 30 % more genes (Fig. 2c), and again on the C1 the protocol performance is even better. Examining the noise in gene expression levels across our samples, we see that genes identified by the original protocol show reduced levels of noise with CEL-Seq2 (Fig. 2d). In particular, for the spike-ins we find lower levels of noise for almost all 92 spiked-in RNA-species (Fig. 2e). Again, to control for differences in sequencing depth, we subsampled 300,000 reads from each sample and obtained similar results (Additional file 2: Figure S2c–g).

### Using CEL-Seq2 to determine cell-cycle associated differences in the transcriptome

**Fig. 3:**
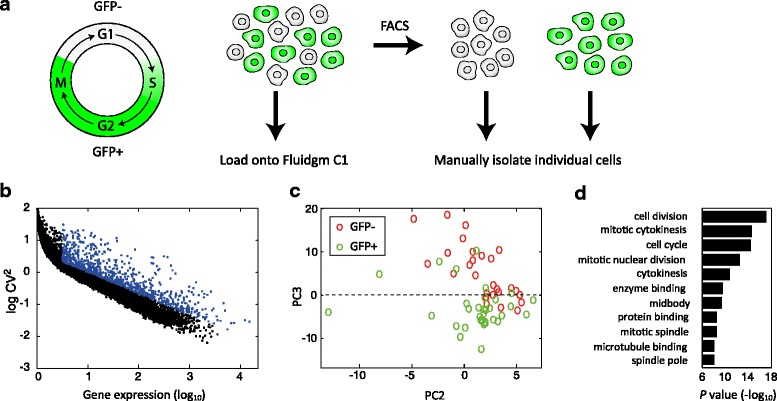
Cell-cycle associated differences in the transcriptome. **a** Experimental system. GFP+ cells are in the S, G2, and M phases of the cell cycle and a growing culture is composed of roughly equal numbers of GFP+ and GFP– cells. Trypsinized cells are either loaded directly onto the Fluidigm C1 with GFP signal observed in the chip, or sorted according to the GFP signal and manually frozen. **b** Coefficient of variation on the C1. Each *dot* indicates a gene’s squared coefficient of variation. **c** PCA of the genes with high coefficient of variation (labeled *blue* in (**b**)). **d** Gene Ontology (GO) terms of differentially expressed genes – PC3 > 0 vs. PC3 < 0 in (**c**)

In order to determine the changes to the transcriptome associated with the cell cycle, we used mouse fibroblast cells carrying a CyclinB1-GFP fusion reporter. These cells are GFP positive (GFP+) during the S, G2, and M phases of the cell cycle, and GFP negative (GFP–) at the G1 phase (Fig. 3a). We used data obtained on the C1, where we can load a mixed population of cells, but determine the GFP status of each before processing the cells. We selected the set of genes that showed high coefficient of variation relative to the mean of expression (Fig. 3b) and performed principal component analysis using these genes. We found that the GFP– and GFP+ cells were well separated (Fig. 3c). When querying for functional enrichments on the set of genes that were differentially expressed across the third principal component, above and below zero, we found that these genes were enriched in cell cycle, cell division, and chromosome segregation (Fig. 3d).

### CEL-Seq2 shows better sensitivity and reproducibility than Smart-Seq

**Fig. 4:**
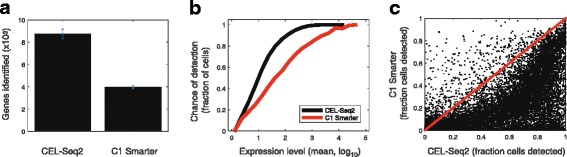
Performance of CEL-Seq2 compared to other methods. **a** Number of genes detected in mouse dendritic cells using CEL-Seq2 and C1 Smarter, as previously described [13]. **b** Chance of detection of transcripts from a particular gene across the two methods. For each gene we computed the average expression across all cells and the fraction of cells in which the expression was detected. For each expression level bin, the average fraction detected was computed. **c** Comparison at the gene-level between the fraction of cells in which expression was detected using the two methods. *Dots* indicate genes

Finally, we sought to directly compare the performance of CEL-Seq2 to another single-cell method by studying an identical cell type. We therefore performed CEL-Seq2 on mouse dendritic cells – formerly the subject of intense analysis using Smart-Seq on the C1 [13]. The dendritic cells were sorted into a 384-well plate containing the CEL-Seq2 primers and dNTPs (see Methods). Following the sort, the plate was frozen to induce lysis with the release of mRNA from the cell and then directly processed – without a clean-up step – using the manual version of the CEL-Seq2 protocol. We found that CEL-Seq2 showed remarkable sensitivity and reproducibly, detecting nearly twice as many genes per cell as Smart-Seq (Fig. 4a). For a given expression level, the chance of detection is higher in CEL-Seq2 (Fig. 4b). Moreover, this pattern is strikingly evident when examining the fraction of cells in which expression is detected for individual genes (Fig. 4c).

While Smart-Seq2 has an improved template switch step relative to Smart-Seq [5], previous work has also shown that C1-based transcriptomics shows increased sensitivity [14], thus making for an appropriate comparison. It is important to note that CEL-Seq2, as a 3′ tag method, differs from Smart-Seq, which produces full-length transcripts. The comparison presented in Fig. 4 is thus complicated by this basic difference. CEL-Seq2 does not provide information on most instances of splicing since it is strongly 3′-biased. However, the sensitivity and ability to individually count transcripts offer a clear advantage for most transcriptomics applications.

## Conclusions

Collectively, CEL-Seq2 benefits from optimized primers, reagents, clean-up, and library preparation step. Together, these modifications greatly improve the quality of the data and make CEL-Seq2 more time- and cost-efficient. Furthermore, our Fluidigm C1-enabled cell-barcoding allows for a single library construction, instead of working individually to set up the library preparation for each cell. Our improvements will also be able to be implemented in the inDrop and Drop-Seq methods [15, 16].

## Methods

### CEL-Seq and CEL-Seq2

CEL-Seq was performed as previously described [6], with the exception that a 5 base UMI was added to the primer and the barcode length was reduced to 6 bases. For CEL-Seq2 the following modifications were introduced (see Additional file 4: Supplementary file 1 for a detailed protocol): (1) A new set of primers was used (Additional file 5: Table S2). Primers are shorter and include an UMI upstream of the barcode. (2) SuperScript® II Double-Stranded cDNA Synthesis Kit is used to convert the mRNA to double stranded DNA. Reagents can be purchased individually, see protocol. (3) Nucleic acid purification steps are performed with RNAClean and AMPure XP beads. (4) The aRNA was converted to cDNA using random priming. A random hexamer was used with a 5′-tail containing the Illumina 3′ adaptor sequence.

### Barcode design

Six nucleotide barcodes were designed so that every pair of barcodes had a Hamming distance of at least 2 bases ensuring that a single sequencing error would not cause the read to be associated with a different sample. Each unique barcode was designed to have a GC content of 33–67 % to avoid low-complexity barcodes which may result in low sequencing quality, and to have the last nucleotide anything other than T. 168 unique barcodes matching these constraints were selected, although larger groups can be constructed.

### Fibroblast culturing

Mouse ear fibroblasts were derived from 1-month-old CyclinB1-GFP mice [12], as previously described [17]. Briefly, a small piece of mouse ear was collected and digested overnight in DMEM containing Collagenase/Dispase. The tissue was then dissociated by gentle pipetting and cells were washed in DMEM/10 % FBS/1 % Pen-Strep/1 % L-Glutamine, pelleted, seeded, and cultured for 2–4 days before sorting on a FACS ARIA (Becton Dickinson).

### Fluidigm C1

Chip priming and cell capture were performed according to the manufacturer’s instructions. After cell capture, CEL-Seq primers were loaded to each of the 96 outlets. The lysis mix was loaded to inlet # 3, RT mix to inlet #4, second strand mix to inlet #7, and IVT mix to inlet # 8, and the CEL-Seq program was run. The resulting aRNA was pooled from all outlets, bead purified, fragmented, and purified again. Library was prepared according to the CEL-Seq2 protocol.

### Sequencing

Paired-end sequencing was performed on the Hiseq 2500 in rapid mode, 15 bases for read 1 (R1), 7 bases for the Illumina index, and 36 bases for read 2 (R2). The data have been deposited under GEO accession number GSE78779.

### Expression analysis pipeline

CEL-Seq reads were processed into an expression matrix using a multistep, parallel computational pipeline within the Galaxy framework [6]. For CEL-Seq2, we developed a new pipeline as a standalone lightweight python program allowing for a faster run-time. The CEL-Seq2 pipeline is compatible also with CEL-Seq reads. The pipeline is distributed under the GPLv3 license allowing others to further customize (https://github.com/yanailab/CEL-Seq-pipeline). The pipeline consists of the following steps: (1) Demultiplexing: using the barcode from R1 we split R2 reads into their original samples creating a separate file for each sample. Since the UMI is also read in R1 we extract it and attach it to the R2 read metadata for downstream analysis. (2) Mapping: using Bowtie2 [18], we map the reads of the different samples in parallel, cutting the analysis time by roughly the number of available cores. (3) Read counting: A modified version of the htseq-count script [19] (https://github.com/yanailab/CEL-Seq-pipeline) that supports the identification and elimination of reads sharing the same UMI to generate an accurate molecule count for each feature. As in Grun et al. [7] we use binomial statistics to convert the number of UMIs into transcript counts. The different steps in the pipeline are wrapped together in a single program with a simple configuration file allowing to control for different run modes. We include the C1 steps as Additional file 6: Supplementary file 2.

### Smart-Seq comparison

Smart-Seq C1 transcriptome data for single mouse dendritic cells were download from GEO under accession number GSE48968.

## Ethics

All animal experiments were performed in accordance with guidelines established by the joint ethics committee (IACUC) of the Hebrew University and Hadassah Medical Center and IACUC protocol number 0612-058-15 at MIT. The Hebrew University is an AAALAC International accredited institute.

## Availability of data and materials

The data have been deposited under GEO accession number GSE78779. Our pipeline is available at https://github.com/yanailab/CEL-Seq-pipeline.

## Additional files

Additional file 1: Figure S1. Optimization of the CEL-Seq protocol. **A** Number of genes obtained from ten replicates of 100 pg RNA performed with each type of primer: the original primer, the original primer with the inclusion of UMI, and the shortened UMI primer. **B** Number of transcripts identified for the two primers containing a UMI. **C** Estimating the efficiency of CEL-Seq using UMIs and ERCC spike-ins. The efficiency is computed as the y-intercept. **D** Side-by-side comparison of column clean-up, bead clean-up, and two RTs relative to CEL-Seq with a UMI primer. **E** Side-by-side comparison of different second-strand synthesis enzymes. The MessageAmp II enzyme was the one used originally. (PDF 519 kb) Additional file 2: Figure S2. Controlling for sequencing depth. Similar results were obtained when subsampling to 300,000 reads. **A**, **B** Same as Fig. 1c, d with the equal subsampling. **C**–**G** Same as Fig. 2 with the equal subsampling. (PDF 2999 kb) Additional file 3: Table S1. Cell capture and GFP+/– signal of cells on the Fluidigm C1. (PDF 55 kb) Additional file 4: Supplementary file 1. The CEL-Seq2 protocol. This file includes the detailed CEL-Seq2 protocol. (DOCX 279 kb) Additional file 5: Table S2. The sequences of the CEL-Seq primers. The 8 base barcodes are as previously published, the 6 base barcodes are listed in the detailed protocol. (PDF 43 kb) Additional file 6: Supplementary file 2. CEL-Seq in C1. The file includes the complete information for performing CEL-Seq on the Fluidigm C1 instrument. (DOCX 17 kb)
