# Supplementary figures and images for: CEL-Seq2: sensitive highly-multiplexed single-cell RNA-Seq
Source: Genome Biol. 2016 Apr 28;17:77. doi: 10.1186/s13059-016-0938-8 (PMC4848782; doi:10.1186/s13059-016-0938-8)

Supplementary Figure 1

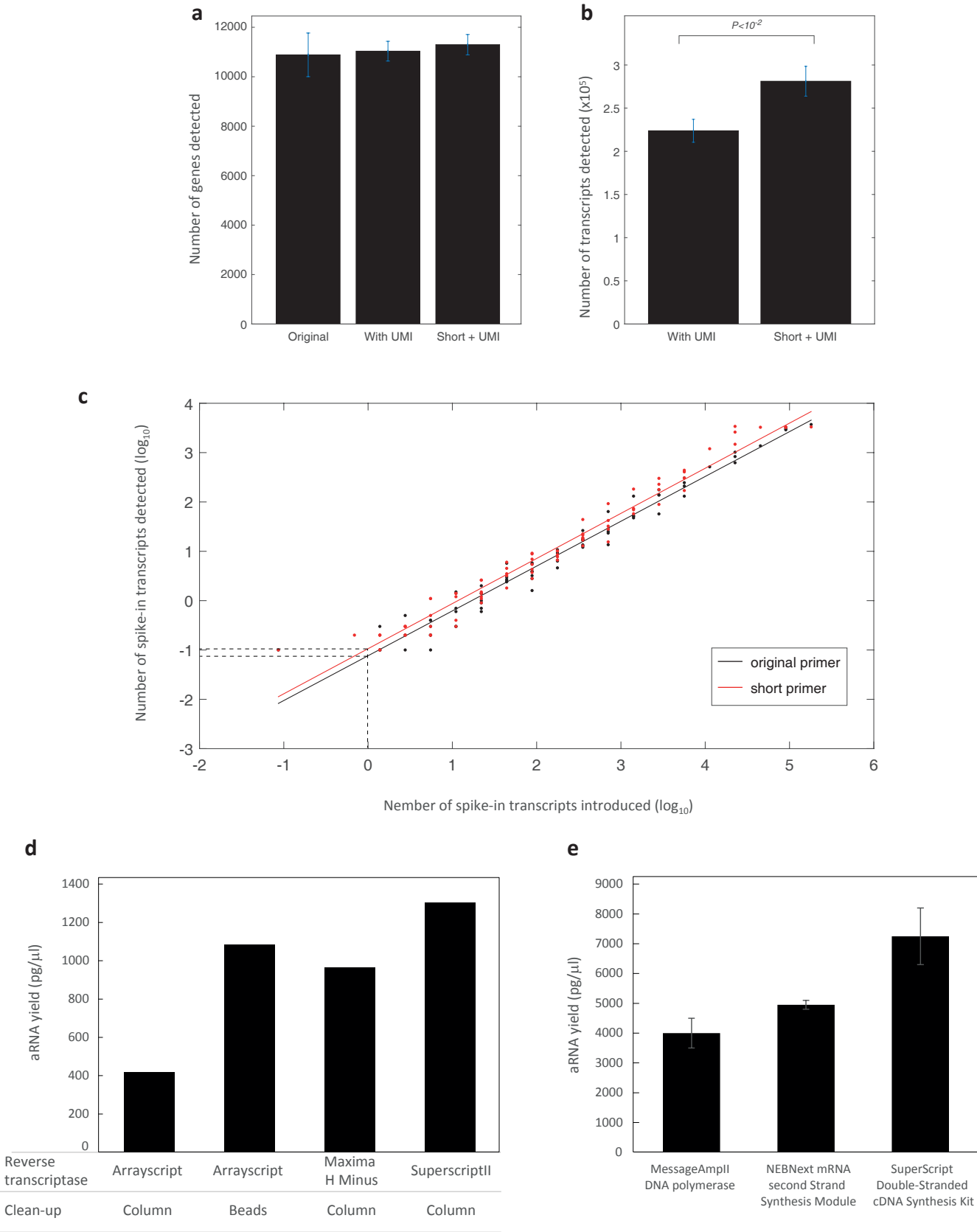

Supplement: Additional file 1: Figure S1. — Optimization of the CEL-Seq protocol. A Number of genes obtained from ten replicates of 100 pg RNA performed with each type of primer: the original primer, the original primer with the inclusion of UMI, and the shortened UMI primer. B Number of transcripts identified for the two primers containing a UMI. C Estimating the efficiency of CEL-Seq using UMIs and ERCC spike-ins. The efficiency is computed as the y-intercept. D Side-by-side comparison of column clean-up, bead clean-up, and two RTs relative to CEL-Seq with a UMI primer. E Side-by-side comparison of different second-strand synthesis enzymes. The MessageAmp II enzyme was the one used originally. (PDF 519 kb) [file 13059_2016_938_MOESM1_ESM.pdf]

Supplementary Figure 2

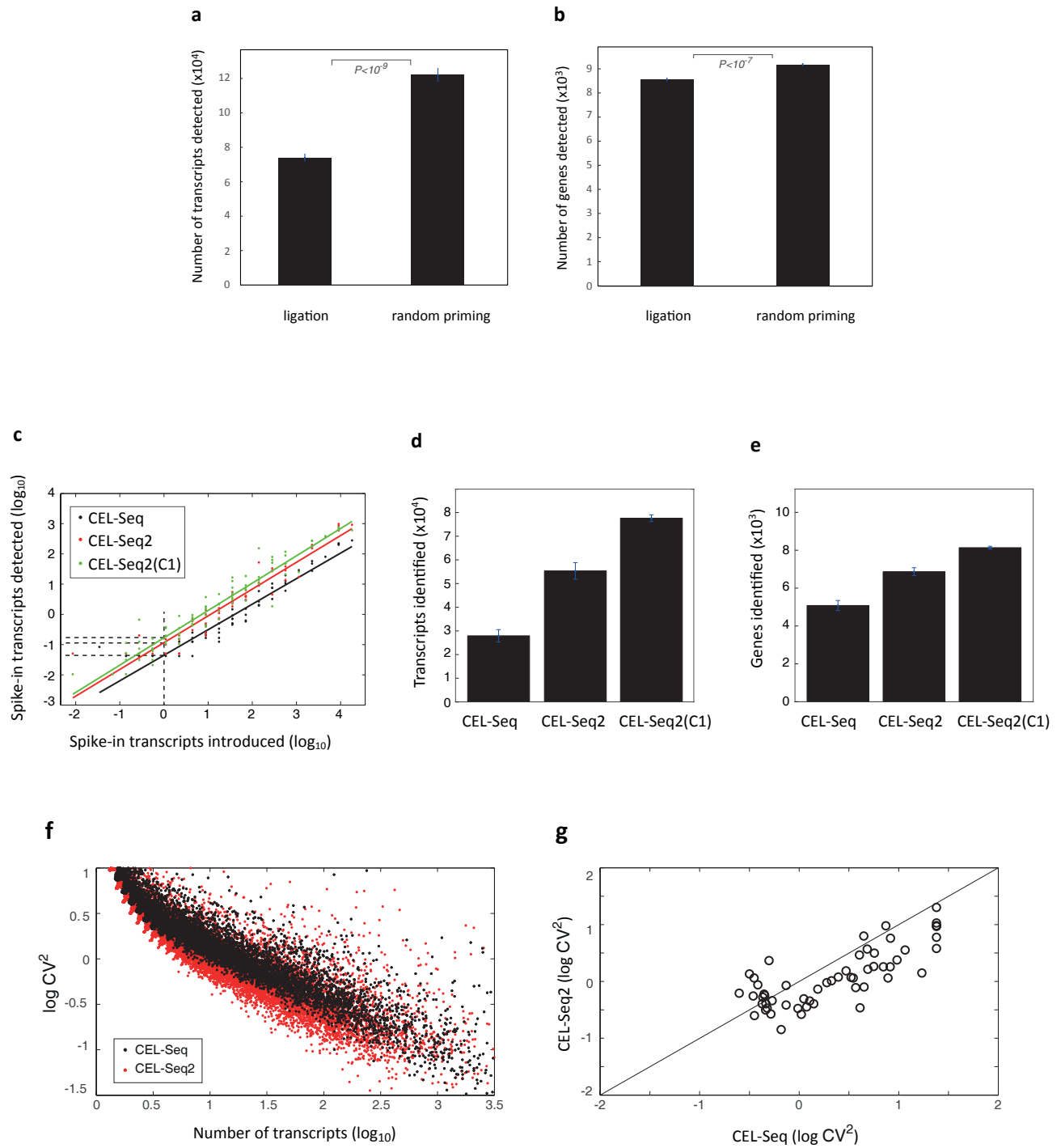

Supplement: Additional file 2: Figure S2. — Controlling for sequencing depth. Similar results were obtained when subsampling to 300,000 reads. A, B Same as Fig. 1c, d with the equal subsampling. C–G Same as Fig. 2 with the equal subsampling. (PDF 2999 kb) [file 13059_2016_938_MOESM2_ESM.pdf]
