# Supplementary material for: CEL-Seq2: sensitive highly-multiplexed single-cell RNA-Seq
Source: Genome Biol. 2016 Apr 28;17:77. doi: 10.1186/s13059-016-0938-8 (PMC4848782; doi:10.1186/s13059-016-0938-8)
Supplement: Additional file 3: Table S1. — Cell capture and GFP+/– signal of cells on the Fluidigm C1. (PDF 55 kb) [file 13059_2016_938_MOESM3_ESM.pdf]

Table S1. Cell capture and GFP+/- signal of cells on the Fluidigm C1.

cell position	barcode	# of cells	GFP
1	33	1	positive
2	17	1	positive
3	1	1	negative
4	41	1	negative
5	25	1	positive
6	9	1	negative
7	34	1	negative
8	18	1	positive
9	2	2	weak
10	42	1	positive
11	26	1	negative
12	10	1	positive
13	35	2	mixed
14	19	1	positive
15	3	1	negative
16	43	2	positive
17	27	1	negative
18	11	1	positive
19	36	1	negative
20	20	2	mixed
21	4	1	?
22	44	~10	mixed
23	28	0	n.a.
24	12	1	positive
25	5	3	mixed
26	21	1	positive
27	37	2	mixed

cell position	barcode	# of cells	GFP
49	1	1	positive
50	17	1	positive
51	33	1	positive
52	9	1	negative
53	25	1	positive
54	41	1	positive
55	2	1	positive
56	18	0	n.a.
57	34	1	negative
58	10	1	negative
59	26	1	positive
60	42	1	positive
61	3	2	positive
62	19	1	weak
63	35	2	mixed
64	11	1	positive
65	27	3	positive
66	43	1	positive
67	4	1	negative
68	20	1	positive
69	36	1	weak
70	12	2	mixed
71	28	1	positive
72	44	1	positive
73	37	1	weak
74	21	2	mixed
75	5	2	negative

28	13	0	n.a.
29	29	1	positive
30	45	1	weak
31	6	1	positive
32	22	1	positive
33	38	1	positive
34	14	1	negative
35	30	1	negative
36	46	2	mixed
37	7	3	mixed
38	23	1	positive
39	39	2	mixed
40	15	1	negative
41	31	1	negative
42	47	2	negative
43	8	1	positive
44	24	1	negative
45	40	1	negative
46	16	1	weak
47	32	2	mixed
48	48	1	negative

76	45	1	positive
77	29	2	mixed
78	13	1	negative
79	38	1	weak
80	22	1	negative
81	6	1	weak
82	46	2	mixed
83	30	1	positive
84	14	1	negative
85	39	1	weak
86	23	1	negative
87	7	1	negative
88	47	2	negative
89	31	1	negative
90	15	1	positive
91	40	1	negative
92	24	1	negative
93	8	1	negative
94	48	1	positive
95	32	1	positive
96	16	1	positive
