# Supplementary material for: CEL-Seq2: sensitive highly-multiplexed single-cell RNA-Seq
Source: Genome Biol. 2016 Apr 28;17:77. doi: 10.1186/s13059-016-0938-8 (PMC4848782; doi:10.1186/s13059-016-0938-8)
Supplement: Additional file 4: — Supplementary file 1. The CEL-Seq2 protocol. This file includes the detailed CEL-Seq2 protocol. (DOCX 279 kb) [file 13059_2016_938_MOESM4_ESM.docx]

CEL-Seq2 Protocol

**Reagents:**

LoBind tubes – 0.5 ml – Eppendorf 022431005

Ultra pure RNase free water

Ethanol

Bioanalyzer kits - Agilent RNA pico kit (5067-1513), high sensitivity DNA kit (5067-4626)

Qubit reagents: dsDNA HS Assay – invitrogen [Q32851](http://products.invitrogen.com/ivgn/en/US/adirect/invitrogen?cmd=catProductDetail&entryPoint=adirect&productID=Q32851) or [Q32854](http://products.invitrogen.com/ivgn/en/US/adirect/invitrogen?cmd=catProductDetail&entryPoint=adirect&productID=Q32854)

For RNA amplification:

ERCC RNA spike-in mix – Ambion 4456740

dNTPs mix 10mM

SuperScript II – Invitrogen 18064-014

RNaseOUT – Invitrogen 10777-019

Second strand buffer – Invitrogen 10812-014

DNA Plymerase I (E. coli) – Invitrogen 18010-025

E. coli DNA ligase – Invitrogen 18052-019

RnaseH (E. coli) – Invitrogen 18021-071

MEGAscript T7 Transcription Kit – Ambion AM1334

ExoSAP-IT For PCR Product Clean-Up – Affymetrix 78200

Fragmentation buffer: 200mM Tris-acetate, pH 8.1, 500 mM KOAc, 150 mM MgOAc

Fragmentation stop buffer: 0.5 M EDTA pH8

AMPure XP beads – Beckman Coulter A63880

RNAClean XP beads - Beckman coulter A63987

Bead binding buffer - 20% PEG8000, 2.5M Nacl

For Library preparation:

SuperScript II – Invitrogen 18064-014

RNaseOUT – Invitrogen 10777-019

AMPure XP beads – Beckman Coulter A63880

Phusion® High-Fidelity PCR Master Mix with HF Buffer – NEB M0531

randomhexRT primer - GCCTTGGCACCCGAGAATTCCANNNNNN

RNA PCR primers (sequences available from Illumina)

Primers re-suspended at 100μM, PCR primers used at 10μM.

**Equipment:**

Thermocycler with lid with adjustable temperature (one that can also fit 0.5 ml PCR tubes is convenient)

Oven (optional)

Magnetic stand (for 0.5 ml tubes)

Qubit® Fluorometer - invitrogen

Bioanalyzer – Agilent

**Primers:**

CEL-Seq primer design: The RT primer was designed with an anchored polyT, a 6 bp unique barcode, a 6 bp UMI (unique molecular identifier), the 5’ Illumina adapter (as used in the Illumina small RNA kit) and a T7 promoter. The barcodes were designed such that each pair is different by at least two nucleotides, so that a single sequencing error will not produce the wrong barcode. Primers are desalted at the lowest possible scale, stock solution 1 μg/μl, working concentration 25ng/μl.

If not using all 48 primers, it is recommended to pool at least 10, and use the following barcodes:

1, 4, 5, 9, 10, 23, 25, 26, 31, 46.

| 1s | GCCGGTAATACGACTCACTATAGGGAGTTCTACAGTCCGACGATCNNNNNNAGACTCTTTTTTTTTTTTTTTTTTTTTTTTV |
| --- | --- |
| 2s | GCCGGTAATACGACTCACTATAGGGAGTTCTACAGTCCGACGATCNNNNNNAGCTAGTTTTTTTTTTTTTTTTTTTTTTTTV |
| 3s | GCCGGTAATACGACTCACTATAGGGAGTTCTACAGTCCGACGATCNNNNNNAGCTCATTTTTTTTTTTTTTTTTTTTTTTTV |
| 4s | GCCGGTAATACGACTCACTATAGGGAGTTCTACAGTCCGACGATCNNNNNNAGCTTCTTTTTTTTTTTTTTTTTTTTTTTTV |
| 5s | GCCGGTAATACGACTCACTATAGGGAGTTCTACAGTCCGACGATCNNNNNNCATGAGTTTTTTTTTTTTTTTTTTTTTTTTV |
| 6s | GCCGGTAATACGACTCACTATAGGGAGTTCTACAGTCCGACGATCNNNNNNCATGCATTTTTTTTTTTTTTTTTTTTTTTTV |
| 7s | GCCGGTAATACGACTCACTATAGGGAGTTCTACAGTCCGACGATCNNNNNNCATGTCTTTTTTTTTTTTTTTTTTTTTTTTV |
| 8s | GCCGGTAATACGACTCACTATAGGGAGTTCTACAGTCCGACGATCNNNNNNCACTAGTTTTTTTTTTTTTTTTTTTTTTTTV |
| 9s | GCCGGTAATACGACTCACTATAGGGAGTTCTACAGTCCGACGATCNNNNNNCAGATCTTTTTTTTTTTTTTTTTTTTTTTTV |
| 10s | GCCGGTAATACGACTCACTATAGGGAGTTCTACAGTCCGACGATCNNNNNNTCACAGTTTTTTTTTTTTTTTTTTTTTTTTV |
| 11s | GCCGGTAATACGACTCACTATAGGGAGTTCTACAGTCCGACGATCNNNNNNAGGATCTTTTTTTTTTTTTTTTTTTTTTTTV |
| 12s | GCCGGTAATACGACTCACTATAGGGAGTTCTACAGTCCGACGATCNNNNNNAGTGCATTTTTTTTTTTTTTTTTTTTTTTTV |
| 13s | GCCGGTAATACGACTCACTATAGGGAGTTCTACAGTCCGACGATCNNNNNNAGTGTCTTTTTTTTTTTTTTTTTTTTTTTTV |
| 14s | GCCGGTAATACGACTCACTATAGGGAGTTCTACAGTCCGACGATCNNNNNNTCCTAGTTTTTTTTTTTTTTTTTTTTTTTTV |
| 15s | GCCGGTAATACGACTCACTATAGGGAGTTCTACAGTCCGACGATCNNNNNNTCTGAGTTTTTTTTTTTTTTTTTTTTTTTTV |
| 16s | GCCGGTAATACGACTCACTATAGGGAGTTCTACAGTCCGACGATCNNNNNNTCTGCATTTTTTTTTTTTTTTTTTTTTTTTV |
| 17s | GCCGGTAATACGACTCACTATAGGGAGTTCTACAGTCCGACGATCNNNNNNTCGAAGTTTTTTTTTTTTTTTTTTTTTTTTV |
| 18s | GCCGGTAATACGACTCACTATAGGGAGTTCTACAGTCCGACGATCNNNNNNTCGACATTTTTTTTTTTTTTTTTTTTTTTTV |
| 19s | GCCGGTAATACGACTCACTATAGGGAGTTCTACAGTCCGACGATCNNNNNNTCGATCTTTTTTTTTTTTTTTTTTTTTTTTV |
| 20s | GCCGGTAATACGACTCACTATAGGGAGTTCTACAGTCCGACGATCNNNNNNGTACAGTTTTTTTTTTTTTTTTTTTTTTTTV |
| 21s | GCCGGTAATACGACTCACTATAGGGAGTTCTACAGTCCGACGATCNNNNNNGTACCATTTTTTTTTTTTTTTTTTTTTTTTV |
| 22s | GCCGGTAATACGACTCACTATAGGGAGTTCTACAGTCCGACGATCNNNNNNGTACTCTTTTTTTTTTTTTTTTTTTTTTTTV |
| 23s | GCCGGTAATACGACTCACTATAGGGAGTTCTACAGTCCGACGATCNNNNNNGTCTAGTTTTTTTTTTTTTTTTTTTTTTTTV |
| 24s | GCCGGTAATACGACTCACTATAGGGAGTTCTACAGTCCGACGATCNNNNNNGTCTCATTTTTTTTTTTTTTTTTTTTTTTTV |
| 25s | GCCGGTAATACGACTCACTATAGGGAGTTCTACAGTCCGACGATCNNNNNNGTTGCATTTTTTTTTTTTTTTTTTTTTTTTV |
| 26s | GCCGGTAATACGACTCACTATAGGGAGTTCTACAGTCCGACGATCNNNNNNGTGACATTTTTTTTTTTTTTTTTTTTTTTTV |
| 27s | GCCGGTAATACGACTCACTATAGGGAGTTCTACAGTCCGACGATCNNNNNNGTGATCTTTTTTTTTTTTTTTTTTTTTTTTV |
| 28s | GCCGGTAATACGACTCACTATAGGGAGTTCTACAGTCCGACGATCNNNNNNACAGTGTTTTTTTTTTTTTTTTTTTTTTTTV |
| 29s | GCCGGTAATACGACTCACTATAGGGAGTTCTACAGTCCGACGATCNNNNNNACCATGTTTTTTTTTTTTTTTTTTTTTTTTV |
| 30s | GCCGGTAATACGACTCACTATAGGGAGTTCTACAGTCCGACGATCNNNNNNACTCTGTTTTTTTTTTTTTTTTTTTTTTTTV |
| 31s | GCCGGTAATACGACTCACTATAGGGAGTTCTACAGTCCGACGATCNNNNNNACTCGATTTTTTTTTTTTTTTTTTTTTTTTV |
| 32s | GCCGGTAATACGACTCACTATAGGGAGTTCTACAGTCCGACGATCNNNNNNACGTACTTTTTTTTTTTTTTTTTTTTTTTTV |
| 33s | GCCGGTAATACGACTCACTATAGGGAGTTCTACAGTCCGACGATCNNNNNNACGTTGTTTTTTTTTTTTTTTTTTTTTTTTV |
| 34s | GCCGGTAATACGACTCACTATAGGGAGTTCTACAGTCCGACGATCNNNNNNACGTGATTTTTTTTTTTTTTTTTTTTTTTTV |
| 35s | GCCGGTAATACGACTCACTATAGGGAGTTCTACAGTCCGACGATCNNNNNNCTAGACTTTTTTTTTTTTTTTTTTTTTTTTV |
| 36s | GCCGGTAATACGACTCACTATAGGGAGTTCTACAGTCCGACGATCNNNNNNCTAGTGTTTTTTTTTTTTTTTTTTTTTTTTV |
| 37s | GCCGGTAATACGACTCACTATAGGGAGTTCTACAGTCCGACGATCNNNNNNCTAGGATTTTTTTTTTTTTTTTTTTTTTTTV |
| 38s | GCCGGTAATACGACTCACTATAGGGAGTTCTACAGTCCGACGATCNNNNNNCTCATGTTTTTTTTTTTTTTTTTTTTTTTTV |
| 39s | GCCGGTAATACGACTCACTATAGGGAGTTCTACAGTCCGACGATCNNNNNNCTCAGATTTTTTTTTTTTTTTTTTTTTTTTV |
| 40s | GCCGGTAATACGACTCACTATAGGGAGTTCTACAGTCCGACGATCNNNNNNCTTCGATTTTTTTTTTTTTTTTTTTTTTTTV |
| 41s | GCCGGTAATACGACTCACTATAGGGAGTTCTACAGTCCGACGATCNNNNNNCTGTACTTTTTTTTTTTTTTTTTTTTTTTTV |
| 42s | GCCGGTAATACGACTCACTATAGGGAGTTCTACAGTCCGACGATCNNNNNNCTGTGATTTTTTTTTTTTTTTTTTTTTTTTV |
| 43s | GCCGGTAATACGACTCACTATAGGGAGTTCTACAGTCCGACGATCNNNNNNTGAGACTTTTTTTTTTTTTTTTTTTTTTTTV |
| 44s | GCCGGTAATACGACTCACTATAGGGAGTTCTACAGTCCGACGATCNNNNNNTGCAACTTTTTTTTTTTTTTTTTTTTTTTTV |
| 45s | GCCGGTAATACGACTCACTATAGGGAGTTCTACAGTCCGACGATCNNNNNNTGCATGTTTTTTTTTTTTTTTTTTTTTTTTV |
| 46s | GCCGGTAATACGACTCACTATAGGGAGTTCTACAGTCCGACGATCNNNNNNTGCAGATTTTTTTTTTTTTTTTTTTTTTTTV |
| 47s | GCCGGTAATACGACTCACTATAGGGAGTTCTACAGTCCGACGATCNNNNNNTGTCACTTTTTTTTTTTTTTTTTTTTTTTTV |
| 48s | GCCGGTAATACGACTCACTATAGGGAGTTCTACAGTCCGACGATCNNNNNNTGTCGATTTTTTTTTTTTTTTTTTTTTTTTV |
| 49s | GCCGGTAATACGACTCACTATAGGGAGTTCTACAGTCCGACGATCNNNNNNTGGTACTTTTTTTTTTTTTTTTTTTTTTTTV |
| 50s | GCCGGTAATACGACTCACTATAGGGAGTTCTACAGTCCGACGATCNNNNNNGACATGTTTTTTTTTTTTTTTTTTTTTTTTV |
| 51s | GCCGGTAATACGACTCACTATAGGGAGTTCTACAGTCCGACGATCNNNNNNGATCACTTTTTTTTTTTTTTTTTTTTTTTTV |
| 52s | GCCGGTAATACGACTCACTATAGGGAGTTCTACAGTCCGACGATCNNNNNNGATCTGTTTTTTTTTTTTTTTTTTTTTTTTV |
| 53s | GCCGGTAATACGACTCACTATAGGGAGTTCTACAGTCCGACGATCNNNNNNGATCGATTTTTTTTTTTTTTTTTTTTTTTTV |
| 54s | GCCGGTAATACGACTCACTATAGGGAGTTCTACAGTCCGACGATCNNNNNNGAGTACTTTTTTTTTTTTTTTTTTTTTTTTV |
| 55s | GCCGGTAATACGACTCACTATAGGGAGTTCTACAGTCCGACGATCNNNNNNAGACAGTTTTTTTTTTTTTTTTTTTTTTTTV |
| 56s | GCCGGTAATACGACTCACTATAGGGAGTTCTACAGTCCGACGATCNNNNNNAGACCATTTTTTTTTTTTTTTTTTTTTTTTV |
| 57s | GCCGGTAATACGACTCACTATAGGGAGTTCTACAGTCCGACGATCNNNNNNAGTGAGTTTTTTTTTTTTTTTTTTTTTTTTV |
| 58s | GCCGGTAATACGACTCACTATAGGGAGTTCTACAGTCCGACGATCNNNNNNAGGAAGTTTTTTTTTTTTTTTTTTTTTTTTV |
| 59s | GCCGGTAATACGACTCACTATAGGGAGTTCTACAGTCCGACGATCNNNNNNAGGACATTTTTTTTTTTTTTTTTTTTTTTTV |
| 60s | GCCGGTAATACGACTCACTATAGGGAGTTCTACAGTCCGACGATCNNNNNNCAACAGTTTTTTTTTTTTTTTTTTTTTTTTV |
| 61s | GCCGGTAATACGACTCACTATAGGGAGTTCTACAGTCCGACGATCNNNNNNCAACCATTTTTTTTTTTTTTTTTTTTTTTTV |
| 62s | GCCGGTAATACGACTCACTATAGGGAGTTCTACAGTCCGACGATCNNNNNNCAACTCTTTTTTTTTTTTTTTTTTTTTTTTV |
| 63s | GCCGGTAATACGACTCACTATAGGGAGTTCTACAGTCCGACGATCNNNNNNCACTCATTTTTTTTTTTTTTTTTTTTTTTTV |
| 64s | GCCGGTAATACGACTCACTATAGGGAGTTCTACAGTCCGACGATCNNNNNNCACTTCTTTTTTTTTTTTTTTTTTTTTTTTV |
| 65s | GCCGGTAATACGACTCACTATAGGGAGTTCTACAGTCCGACGATCNNNNNNCAGAAGTTTTTTTTTTTTTTTTTTTTTTTTV |
| 66s | GCCGGTAATACGACTCACTATAGGGAGTTCTACAGTCCGACGATCNNNNNNCAGACATTTTTTTTTTTTTTTTTTTTTTTTV |
| 67s | GCCGGTAATACGACTCACTATAGGGAGTTCTACAGTCCGACGATCNNNNNNTCACCATTTTTTTTTTTTTTTTTTTTTTTTV |
| 68s | GCCGGTAATACGACTCACTATAGGGAGTTCTACAGTCCGACGATCNNNNNNTCACTCTTTTTTTTTTTTTTTTTTTTTTTTV |
| 69s | GCCGGTAATACGACTCACTATAGGGAGTTCTACAGTCCGACGATCNNNNNNTCCTCATTTTTTTTTTTTTTTTTTTTTTTTV |
| 70s | GCCGGTAATACGACTCACTATAGGGAGTTCTACAGTCCGACGATCNNNNNNTCCTTCTTTTTTTTTTTTTTTTTTTTTTTTV |
| 71s | GCCGGTAATACGACTCACTATAGGGAGTTCTACAGTCCGACGATCNNNNNNTCTGTCTTTTTTTTTTTTTTTTTTTTTTTTV |
| 72s | GCCGGTAATACGACTCACTATAGGGAGTTCTACAGTCCGACGATCNNNNNNGTCTTCTTTTTTTTTTTTTTTTTTTTTTTTV |
| 73s | GCCGGTAATACGACTCACTATAGGGAGTTCTACAGTCCGACGATCNNNNNNGTTGAGTTTTTTTTTTTTTTTTTTTTTTTTV |
| 74s | GCCGGTAATACGACTCACTATAGGGAGTTCTACAGTCCGACGATCNNNNNNGTTGTCTTTTTTTTTTTTTTTTTTTTTTTTV |
| 75s | GCCGGTAATACGACTCACTATAGGGAGTTCTACAGTCCGACGATCNNNNNNGTGAAGTTTTTTTTTTTTTTTTTTTTTTTTV |
| 76s | GCCGGTAATACGACTCACTATAGGGAGTTCTACAGTCCGACGATCNNNNNNACAGACTTTTTTTTTTTTTTTTTTTTTTTTV |
| 77s | GCCGGTAATACGACTCACTATAGGGAGTTCTACAGTCCGACGATCNNNNNNACAGGATTTTTTTTTTTTTTTTTTTTTTTTV |
| 78s | GCCGGTAATACGACTCACTATAGGGAGTTCTACAGTCCGACGATCNNNNNNACCAACTTTTTTTTTTTTTTTTTTTTTTTTV |
| 79s | GCCGGTAATACGACTCACTATAGGGAGTTCTACAGTCCGACGATCNNNNNNACCAGATTTTTTTTTTTTTTTTTTTTTTTTV |
| 80s | GCCGGTAATACGACTCACTATAGGGAGTTCTACAGTCCGACGATCNNNNNNACTCACTTTTTTTTTTTTTTTTTTTTTTTTV |
| 81s | GCCGGTAATACGACTCACTATAGGGAGTTCTACAGTCCGACGATCNNNNNNCTCAACTTTTTTTTTTTTTTTTTTTTTTTTV |
| 82s | GCCGGTAATACGACTCACTATAGGGAGTTCTACAGTCCGACGATCNNNNNNCTTCACTTTTTTTTTTTTTTTTTTTTTTTTV |
| 83s | GCCGGTAATACGACTCACTATAGGGAGTTCTACAGTCCGACGATCNNNNNNCTTCTGTTTTTTTTTTTTTTTTTTTTTTTTV |
| 84s | GCCGGTAATACGACTCACTATAGGGAGTTCTACAGTCCGACGATCNNNNNNCTGTTGTTTTTTTTTTTTTTTTTTTTTTTTV |
| 85s | GCCGGTAATACGACTCACTATAGGGAGTTCTACAGTCCGACGATCNNNNNNTGAGTGTTTTTTTTTTTTTTTTTTTTTTTTV |
| 86s | GCCGGTAATACGACTCACTATAGGGAGTTCTACAGTCCGACGATCNNNNNNTGAGGATTTTTTTTTTTTTTTTTTTTTTTTV |
| 87s | GCCGGTAATACGACTCACTATAGGGAGTTCTACAGTCCGACGATCNNNNNNTGTCTGTTTTTTTTTTTTTTTTTTTTTTTTV |
| 88s | GCCGGTAATACGACTCACTATAGGGAGTTCTACAGTCCGACGATCNNNNNNTGGTTGTTTTTTTTTTTTTTTTTTTTTTTTV |
| 89s | GCCGGTAATACGACTCACTATAGGGAGTTCTACAGTCCGACGATCNNNNNNTGGTGATTTTTTTTTTTTTTTTTTTTTTTTV |
| 90s | GCCGGTAATACGACTCACTATAGGGAGTTCTACAGTCCGACGATCNNNNNNGAAGACTTTTTTTTTTTTTTTTTTTTTTTTV |
| 91s | GCCGGTAATACGACTCACTATAGGGAGTTCTACAGTCCGACGATCNNNNNNGAAGTGTTTTTTTTTTTTTTTTTTTTTTTTV |
| 92s | GCCGGTAATACGACTCACTATAGGGAGTTCTACAGTCCGACGATCNNNNNNGAAGGATTTTTTTTTTTTTTTTTTTTTTTTV |
| 93s | GCCGGTAATACGACTCACTATAGGGAGTTCTACAGTCCGACGATCNNNNNNGACAACTTTTTTTTTTTTTTTTTTTTTTTTV |
| 94s | GCCGGTAATACGACTCACTATAGGGAGTTCTACAGTCCGACGATCNNNNNNGACAGATTTTTTTTTTTTTTTTTTTTTTTTV |
| 95s | GCCGGTAATACGACTCACTATAGGGAGTTCTACAGTCCGACGATCNNNNNNGAGTTGTTTTTTTTTTTTTTTTTTTTTTTTV |
| 96s | GCCGGTAATACGACTCACTATAGGGAGTTCTACAGTCCGACGATCNNNNNNGAGTGATTTTTTTTTTTTTTTTTTTTTTTTV |

**Single cell isolation**:

Individual cells (so far we’ve worked with *C. elegans* blastomeres or trypsinised tissue culture cells) are transferred with a micro-pipette into a 0.5µl drop of appropriate buffer (egg salts or PBS) placed on the cap of a 0.5 ml LoBind Eppendorf tube. Location of cell should be marked. Excess liquid is aspirated off leaving only the cell behind, and tube is frozen in liquid nitrogen. Samples are stored at -80°C.

Alternatively, cells can be sorted into plates prepared ahead of time, containing 1.2 μl primer mix, a different primer in each well. Plates are frozen in -80°C until ready to proceed.

**RNA Amplification:**

Prepare primer mix (for each different primer used):

Primer (25ng/μl) 1μl

ERCC Spike-in Xμl

dNTPs 10mM 0.5μl

water Yμl

6μl

Spike-in dilution should be appropriate for sample size – see protocol of ERCC RNA spike in mix. For single cells we add the equivalent of 1ul of spike-in at 1:1,000,000 dilution.

Breaking cell open and annealing with primer:

- Add 1.2μl primer mix to marked location of single cell on cap of tube (Keep cell frozen until adding the primer mix, handle up to 12 cells in parallel).
- Incubate 2.5 min. at 65^o^C (with lid of thermal cycler set to 65^o^C).
- Brief spin down.
- Incubate for an additional 2.5 min. at 65^o^C.
- Move immediately to ice.
- Spin at maximal speed for a few seconds to collect as many droplets as possible before next step, and then return to ice.

For sorted cells in a plate – remove from -80°C, and go straight to a 5 minute incubation at 65°C. Move immediately to ice. Spin at maximal speed for a few seconds to collect as many droplets as possible before next step, and then return to ice.

For using clean RNA – see note.

RT reaction:

- Add 0.8μl of the following mix to each reaction:

First Strand buffer 0.4μl

DTT 0.1M 0.2μl

RNase Inhibitor 0.1μl

SuperscriptII 0.1μl

- Incubate 1hr at 42^o^C (in hybridization oven, or thermal cycler with lid at 50 ^o^C)
- Heat inactivate 10min at 70 ^o^C

Second strand reaction:

- Move previous step to ice so it cools below 16^o^C.
- Add 10μL of the following mix to each reaction tube:

DDW 7μl

Second strand buf. 2.31μl

dNTP 0.23μl

ligase 0.08μl

E. coli DNA Pol 0.3μl

RNaseH 0.08μl

Flick and spin samples (at maximal speed for a few seconds).

(At this point samples are already barcoded, so if all samples are going to the same IVT, a single tip can be used.)

- Incubate at 16^o^C for 2hr (in thermal cycler with unheated or open lid).

cDNA cleanup:

- Prewarm AMPure XP beads to room temperature.
- Pool all cells that are to go to same IVT. Should have ~12μl from each cell.
- Vortex AMPure XP Beads until well dispersed, then add to 100μl pooled sample 20μl beads and 100μl bead buffer (for single/few reaction just add 1.2 volume beads).
- Incubate at room temperature for 15 min.
- Place on magnetic stand for at least 5 min, until liquid appears clear.
- Remove and discard 200μl of the supernatant.
- Add 200μl freshly prepared 80% EtOH.
- Incubate at least 30 seconds, then remove and discard supernatant without disturbing beads.
- Add 200μl freshly prepared 80% EtOH
- Incubate at least 30 seconds, then remove and discard supernatant without disturbing beads.
- Air dry beads for 15 min, or until completely dry.
- Resuspend with 6.4μl water. Pipette entire volume up and down ten times to mix thoroughly.
- Incubate at room temperature for 2 min.
- Go straight to IVT

Cleanup can be replaced by heat inactivated for 20 minutes at 65 ^o^C.

IVT (Ambion kit):

- Prepare the following mix and add 9.6μl per tube (scale up if cDNA volume is more than 6.4)

A 1.6μl

G 1.6μl

C 1.6μl

U 1.6μl

10xT7 buffer 1.6μl

T7 enzyme 1.6μl

- Incubate in a thermal cycler at 37^o^C for 13 hrs, with lid at 70^o^C. Set cycler to go to 4^o^C at end of incubation. aRNA (amplified RNA) is stable for at least several hours.

EXO-SAP treatment (to remove primers):

- Add 6 μl enzyme
- Incubate at 15 minutes at 37 ^o^C

RNA fragmentation:

- Mix the following on ice:

aRNA 22μl

Fragmentation buffer 5.5μl

- Incubate for 3 min. at 94^o^C.
- Immediately move to ice and add 2.75μl fragmentation stop buffer.

Remove beads:

- Place on magnetic stand for at least 5 min, until liquid appears clear.
- Transfer the supernatant to new tube.

aRNA cleanup:

- Prewarm RNAClean XP beads to room temperature.
- Vortex RNAClean XP beads until well dispersed, add to sample 55μl beads. (1.8 volumes)
- Incubate at room temperature for 10 min.
- Place on magnetic stand for at least 5 min, until liquid appears clear.
- Remove and discard ~80μl of the supernatant.
- Add 200μl freshly prepared 70% EtOH.
- Incubate at least 30 seconds, then remove and discard supernatant without disturbing beads.
- Repeat wash two more times.
- Air dry beads for 15 min, or until completely dry.
- Resuspend with 7μl water. Pipette entire volume up and down ten times to mix thoroughly.
- Incubate at room temperature for 2 min.
- Place on magnetic stand for 5 min, until liquid appears clear.
- Transfer supernatant to new tube.

Stopping point: Samples can be kept at -80^o^C

Check aRNA amount and quality:

Optional – when starting with few or very small cells aRNA is not always visible, but this is not necessarily an indication of failed amplification. Go straight to library preparation, resuspend beads above in 5.5μl instead of 7μl.

- Load 1μl onto Bioanalyzer RNA pico chip after heating an aliquot of the sample to 70^o^ for 2 min.
- When starting the IVT with ~0.1ng total RNA, the expected yield is 500-1000 pg/μl. Size distribution should peak at ~500 bp (See Bioanalyzer plot for example).

**
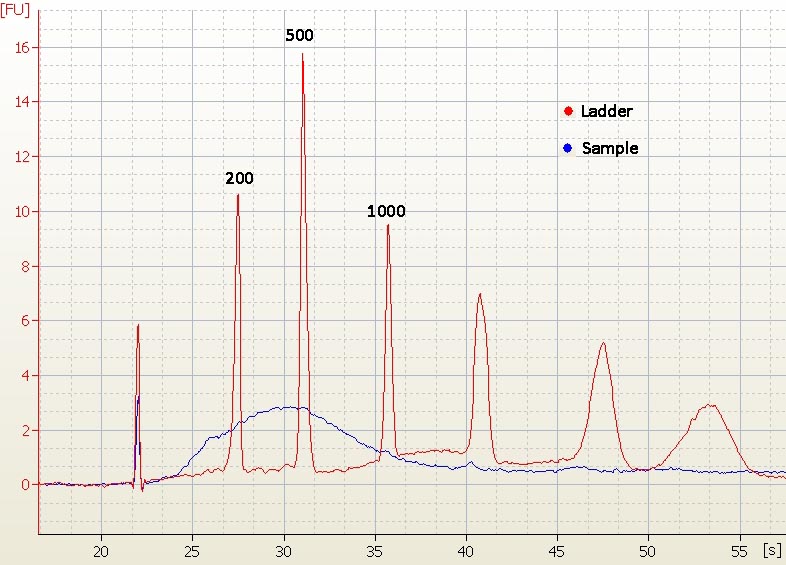
**

**Library preparation:**

RT reaction:

To 5 μl RNA add 1 μl randomhexRT primer and 0.5 μl dNTPs .

Incubate 5min at 65 ^o^C, quick chill on ice.

- Add 4μl of the following mix at room temperature to each reaction:

First Strand buffer 2μl

DTT 0.1M 1μl

RNaseOUT 0.5μl

SuperscriptII 0.5μl

Incubate 10 min at 25 ^o^C.

Incubate 1hr at 42^o^C (in hybridization oven, or pre heated thermal cycler with lid at 50 ^o^C)

PCR amplification:

(Half a PCR reaction is enough, this way can start with low number of cycles and repeat with more cycles if needed)

To each reverse transcription reaction add 38μl of the following mix:

- Ultra-pure Water 11μL
- PCR mix 25μL
- RNA PCR Primer (RP1, from Illumina kit) 2μL

To each reaction add 2μl of a uniquely indexed RNA PCR Primer (RPIX, sequences from Illumina kit, choose balanced primers according to Illumina's pooling guide)

Amplify the tube in the thermal cycler using the following PCR cycling conditions:

- 30 seconds at 98°C
- 11 cycles of:
- 10 seconds at 98°C
- 30 seconds at 60°C
- 30 seconds at 72°C
- 10 minutes at 72°C
- Hold at 4°C

Can go up to 15 cycles if necessary, if aRNA concentration was low.

Stopping point: samples can be kept at -20^o^C.

Bead Cleanup of PCR products – Repeat 1:

- Prewarm beads to room temperature.
- Vortex AMPure XP Beads until well dispersed, then add 50μl to the 50μl PCR reaction (or 25 μl if half a PCR reaction was performed). Mix entire volume up ten times to mix thoroughly.
- Incubate at room temperature for 15 min.
- Place on magnetic stand for at least 5 min, until liquid appears clear.
- Remove and discard 95μl (or 45 μl ) of the supernatant.
- Add 200μl freshly prepared 80% EtOH.
- Incubate at least 30 seconds, then remove and discard supernatant without disturbing beads.
- Add 200μl freshly prepared 80% EtOH
- Incubate at least 30 seconds, then remove and discard supernatant without disturbing beads.
- Air dry beads for 15 min, or until completely dry.
- Resuspend with 25μl water. Pipette entire volume up and down ten times to mix thoroughly.
- Incubate at room temperature for 2 min.
- Place on magnetic stand for 5 min, until liquid appears clear.
- Transfer 25μl of supernatant to new tube.

Bead Cleanup of PCR products – Repeat 2:

Repeat as above, adding 25μl beads and eluting in 10μl water at the end, transferring 10μl to a new tube.

Check library amount and quality:

Check concentration of DNA by Qubit, 1μl should be enough to measure using the high sensitivity reagent; expected concentration is at least ~1ng/μl.

Run 1μl of each sample on Bioanalyzer using a high sensitivity DNA chip to see size distribution. Expected peak at 200-400bp (See Bioanalyzer plot for example).


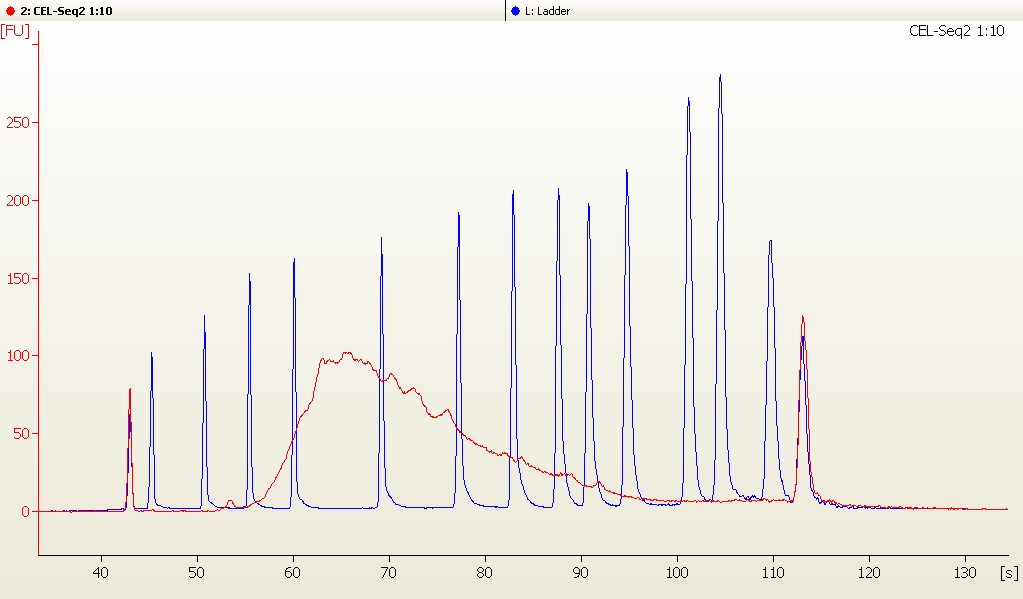


Concentration to be loaded for sequencing should be calibrated by the sequencing facility. We are using 8pM with HiSeq high throughput v.3 reagents, or 12pM on MiSeq and HiSeq rapid mode. Paired end sequencing is performed, 15 bases for read 1, 7 for the illumine index (when needed) and 36 bases for read 2. Throughout the Illumina sequencing the libraries should be considered Small-RNA libraries. For example, in Illumina Rehyb. kits, some Illumina kits are not for Small-RNA.

Currently, CEL-Seq libraries are not compatible with Illumina HiSeq high throughput v.4 reagents.

**Notes:**

CEL-Seq can be used to amplify clean RNA;

- Prepare an RNA/primer/spike-in/dNTPs. mix. Amount of primer and dNTPs as above, appropriate amount of spike-in can be added to the clean RNA or directly to the sample before RNA prep.
- Transfer 1.2μl of mix to new tube (left over mix can be kept at -70^o^C).
- Incubate for 5 min. at 65^o^C.
- Move immediately to ice.
- Spin at maximal speed for a few seconds to collect as many droplets as possible before next step, and then return to ice.

Continue by adding RT mix as in the main protocol.
