# Supplementary material for: CEL-Seq2: sensitive highly-multiplexed single-cell RNA-Seq
Source: Genome Biol. 2016 Apr 28;17:77. doi: 10.1186/s13059-016-0938-8 (PMC4848782; doi:10.1186/s13059-016-0938-8)
Supplement: Additional file 5: Table S2. — The sequences of the CEL-Seq primers. The 8 base barcodes are as previously published, the 6 base barcodes are listed in the detailed protocol. (PDF 43 kb) [file 13059_2016_938_MOESM5_ESM.pdf]

**Table S2.** The sequences of the CEL-Seq primers. The 8 base barcodes are as previously published, the 6 base barcodes are listed in the detailed protocol.

| | |
|---|---|
| CEL-Seq | CGATTGAGGCCGGTAATACGACTCACTATAGGGGTTCAGAGTTCTACAGTCCGACGATC[8 base barcode]TTTTTTTTTTTTTTTTTTTTTTTTV |
| CEL-Seq + UMI | CGATTGAGGCCGGTAATACGACTCACTATAGGGGTTCAGAGTTCTACAGTCCGACGATCNNNNN[6 base barcode]TTTTTTTTTTTTTTTTTTTTTTTTV |
| CEL-Seq2 | GCCGGTAATACGACTCACTATAGGGAGTTCTACAGTCCGACGATCNNNNNN[6 base barcode]TTTTTTTTTTTTTTTTTTTTTTTTV |
| Library RT primer | GCCTTGGCACCCGAGAATTCCANNNNNN |
