# Supplementary material for: CEL-Seq2: sensitive highly-multiplexed single-cell RNA-Seq
Source: Genome Biol. 2016 Apr 28;17:77. doi: 10.1186/s13059-016-0938-8 (PMC4848782; doi:10.1186/s13059-016-0938-8)
Supplement: Additional file 6: — Supplementary file 2. CEL-Seq in C1. The file includes the complete information for performing CEL-Seq on the Fluidigm C1 instrument. (DOCX 17 kb) [file 13059_2016_938_MOESM6_ESM.docx]

**Purchases**

Life Technologies

MessageAmp™ II aRNA Amplification Kit

Ambion AM1751

Life Technologies

ERCC RNA Spike-In Mix

Ambion 4456740

Life Technologies

SUPERase•In™ RNase Inhibitor (20 U/μL)

Ambion AM2694 or AM2696

Thermo Scientific

dNTP Mix, 25mM each

FERR1121

**Stocks**

5× Lysis Buffer

2.5% NP-40 (10%, Thermo Scientific 28324)

250 mM Tris-HCl, pH 8.4

5 mM EDTA

CEL-Seq Primers Stock

Plate of 96 primers each at a concentration of 25ng/ul in TE

**C_1_ Mixes**

Lysis Mix (1000 µL)

280 µL 5× Lysis Buffer

7 µL 20 U/μL SUPERase• In

5.5 µL 1:100 dilution of ERCC RNA Spike-In Mix

70 µL C_1_™ Loading Reagent (Fluidigm 100-5170)

638 µL H_2_O

9 µL Lysis Mix to Inlet #3

Diluted CEL-Seq Primers

Dispense 8 µL Lysis Mix to each of 96 wells

Add 2 µL CEL-Seq Primer Stock to each well

3 µL Diluted CEL-Seq Primer to each of 96 Outlets

RT Mix (10 µL)

3.5 µL 10× First Strand Buffer (from MessageAmp kit)

1.75 µL RNase Inhibitor (from MessageAmp kit)

1.75 µL Array Script Reverse Transcriptase (from MessageAmp kit)

3 µL of the following mix:

1.4 µL 25 mM dNTPs

1 µL C_1_™ Loading Reagent (Fluidigm 100-5170)

3.6 µL H_2_O

9 µL RT Mix to Inlet #4

2^nd^ Strand Mix (40.5 µL)

5 µL 10× Second Strand Buffer (from MessageAmp kit)

2 µL dNTP Mix (from MessageAmp kit)

1 µL DNA Polymerase (from MessageAmp kit)

0.5 µL RNase H (from MessageAmp kit)

2 µL C_1_™ Loading Reagent (Fluidigm 100-5170)

30 µL H_2_O

24 µL 2^nd^ Strand Mix to Inlet #7

IVT Mix (26.9 µL)

6 µL T7 10× Reaction Buffer (from MessageAmp kit)

13.5 µL rNTP (6ul of each, speedvaced down to 13.5ul)

6 µL T7 Enzyme Mix (from MessageAmp kit)

1.36 µL C_1_™ Loading Reagent (Fluidigm 100-5170)

24 µL IVT Mix to Inlet #8

**C_1_ Steps**

1. 96 Diluted CEL-Seq Primers to chamber 1 from the 96 Outlet wells
2. Lysis Mix to chambers 0+1+2 from Inlet #3

65°C, 5 min (300 sec)

10°C, 1 min (60 sec)

1. RT Mix to chambers 0+1+2+3 from Inlet #4

42°C, 120 min (7200 sec)

10°C, 1 min (60 sec)

1. 2^nd^ Strand Mix to chambers 0+1+2+3+4 from Inlet #7

16°C, 120 min (7200 sec)

65°C, 20 min (1200 sec)

10°C, 1 min (60 sec)

1. IVT Mix to chambers 0+1+2+3+4+5 from Inlet #8

37°C, 12 hr (12× 3600 sec)

10°C, 1 min (60 sec)

1. Harvest

**aRNA Clean up**

1. Pool 5µl of each of the harvested aRNA libraries (~480µl total)
2. Add 1.8× volumes of RNAClean XP beads to the harvested aRNA. Mix well until the liquid appears homogeneous. Incubate for 10 min at room temperature.
3. Bind beads on a magnetic stand for 5 min.
4. Discard the supernatant.
5. Wash 3 times with 70% ethanol.
6. Air dry for 10 min or until completely dry.
7. Resuspend the beads with 16 µl RNase-free DDW. Incubate in room temperature for 2 min.
8. Bind beads on a magnetic stand for 5 min.
9. Transfer the eluted aRNA to a new Eppendorf, continue with ExoSAP treatment, as in regular CEL-Seq2 protocol.
